# Ultra-low loss silicon nitride becomes even cooler

**DOI:** 10.1038/s41377-024-01576-1

**Published:** 2024-09-05

**Authors:** Dawn T. H. Tan, Xavier X. Chia

**Affiliations:** 1 Photonics Devices and Systems Group, Singapore University of Technology and Design, Singapore, Singapore; 2 Institute of Microelectronics, Agency for Science Technology and Research (A*STAR), Singapore, Singapore

**Keywords:** Optical physics, Optical physics

## Abstract

Ultra-low loss silicon nitride realized using deuterated precursors and low thermal budgets well within backend-of-line CMOS processing may accelerate widespread proliferation of their use.

The use of silicon nitride in photonic integrated circuits (PICs) has accelerated greatly from the ultra-low losses availed by low pressure chemical vapor deposition (LPCVD) grown, high temperature annealed films. Some of the first notable demonstrations of their optical properties use in PICs came at a time when silicon was languishing from community wide recognition that their nonlinear optical performance at telecommunications wavelengths was poor^1^. The characterization of fundamental optical quantities associated with SiN was an important first step^2,3^. Sub-dB/cm propagation losses were sufficient for resonator quality factors on the hundreds of thousands, paving the way for the demonstration of optical parametric oscillation in SiN-based microresonators^4^. SiN-based photonic integrated circuits have since experienced significant advancements^5–8^, in applications driven by frequency combs such as, high-speed data transmission^9–11^, frequency synthesis^12^ as well as optical parametric amplifiers^13^ and laser stabilization^14^.

Commensurate with the plethora of applications SiN PICs have, extensive efforts and creativity from various teams to systematically eliminate sources of loss have been undertaken. PIC losses impact threshold powers in frequency combs, conversion efficiencies in harmonic generation, impact thermorefractive noise in microcavities and may serve as the determinant for successful data detection at the end of a transceiver link. The fundamental origin of loss in chemical vapor deposition (CVD) grown SiN films stems from Si-H bonds (and N-H bonds) which form from SiH_4_, SiH_2_Cl_2_ and NH_3_ precursor gases used during deposition. To this end, the vast majority of ultra-low loss SiN today utilizes LPCVD (typically 800 °C) and post deposition annealing (typically 1050–1200 °C for several hours)^15,16^. Such high temperature processing is adopted to drive out Si-H bonds, the third vibrational overtone of which resides at the telecommunications C-band and causes material absorption^17^. In addition, stress associated with LPCVD requires film stress management which may take the form of adoption of trenches^15^ and multi-step deposition^16^.

Recently, Bose et al. report the use of SiD_4_ gas in low temperature (250 °C) grown, anneal-free SiN for PICs with ultra-low losses^18^. SiD_4_ utilizes deuterium, an isotope of hydrogen, thus resulting in films which possess Si-D bonds in place of Si-H bonds. Importantly, the SiO_2_ upper cladding used in this work is also deposited using SiD_4_ gas, especially important for the devices with low modal confinement since >50% of potential absorption could occur in the cladding. The replacement of Si-D bonds with Si-H bonds in CVD grown films has been reported to reduce losses via a shift in the absorption overtone from the 1.5 μm region to the 2 μm region in both SiN, and silicon-rich nitride where films are engineered to have a higher silicon content to increase the Kerr nonlinearity^19–23^. This substitution has profound implications, having been previously shown to obviate the need for high temperature growth or post deposition annealing for eliminating the main source of loss in CVD grown SiNx. Fortuitously, low temperature CVD grown films may possess less stress, further reducing process complexity. Remarkably, they demonstrate the lowest loss, anneal free SiN films to date, reporting 1.77 dB/m and 8.66 dB/m loss for low confinement and high confinement waveguides respectively. The development of ultra-low loss devices with low modal confinement and high modal confinement is salient since each of the two categories has distinct applications.

Figure 1 shows the delineation of low confinement and high confinement devices and their potential applications. In general, low-loss, high confinement SiN devices have been more widely studied with more demonstrations of applications. High confinement SiN devices have the appropriate geometrical attributes to confer anomalous dispersion, important for the generation of frequency combs and facilitating nonlinear effects such as cascaded four wave mixing, dispersion wave formation, optical soliton formation and fission, all of which may cumulatively augment the formation of broadband supercontinuum. High modal confinement implies that the majority of the optical mode interacts with the SiN core as opposed to the SiO_2_ cladding, also important for nonlinear processes where nonlinear phase acquisition will take place more efficiently in SiN than SiO_2_, given an order of magnitude higher Kerr nonlinearity in the former. In addition, the nonlinear parameter scales inversely with the effective mode area, which is smaller in high confinement waveguides. Though less widely studied, low confinement SiN enables important applications as well. The effective mode area in low confinement SiN waveguides may be an order of magnitude larger than in high confinement SiN waveguides. For example, the effective modal area of the low confinement (TM mode) waveguide and high confinement waveguide (TE mode) used by Bose et al. is 20 μm^2^ and 1.3 μm^2^. They are thus less susceptible to roughness on the sides and tops of the waveguides and therefore have the potential to have significantly lower losses. Given that a large extent of the low confinement SiN’s optical mode resides in the cladding, the deuterated process for the SiO_2_ implemented in the process is also important to yield the lowest losses.

**Fig. 1:**
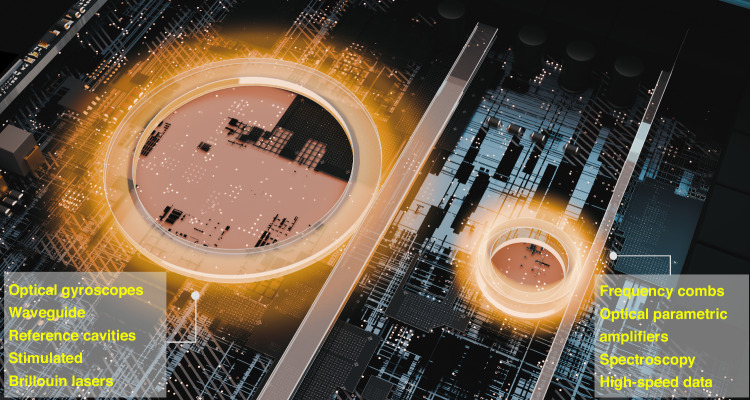
Low confinement and high confinement SiN devices on a chip. Each of the two classes of devices has different optical properties and serves different types of applications

In Bose et al.’s article, they characterize the propagation losses and quality factor of the quasi-TM mode of the low confinement waveguide, a judicious choice given that roughness on the deposited SiN surface is smaller than the etched sidewalls. The highest intrinsic quality factor and lowest propagation losses achieved using these microring resonators are 14.9 million and 1.77 dB/m respectively. Laser stabilization through Pound-Drevor-Hall locking to the SiN resonator results in a remarkable four order of magnitude reduction in frequency noise. The optical properties of the ultra-low loss, low confinement devices make them well suited for future chip-scale implementations of gyroscopes and stimulated Brillouin lasers.

In the same vein, ultra-low losses in high confinement SiN devices using the low temperature deuterated silane process enable a whole suite of other distinct applications (see Fig. 1). Amongst these, Bose et al. demonstrate the generation of supercontinuum over two octaves in waveguides and observations of optical parametric oscillation at a threshold power of ~16.7 mW using a ring resonator possessing an intrinsic quality factor of 4.03 million. The generation of soliton states, coveted for their coherence, low phase noise and high spectral density for modulation of data, at even lower powers and temperatures than previously possible, would be the natural next step to fully exploit the ultra-low loss, thick SiN developed in this work.

The implications of low temperature, ultra-low loss silicon nitride go beyond specific applications, reducing cost and process complexity, potentially impacting how optoelectronic circuits may be manufactured and possibly opening up new classes of circuits which can be manufactured. Consider the typical process flow of an electronics chip which first undergoes frontend-of-line CMOS processing. Here, transistors and electrical interconnects are defined, encompassing processes such as photolithography, etching, ion implantation, gate oxide and electrode formation and source/drain implantation. Thereafter, low k- and copper-based metal interconnects are processed. Considering the process flow, if the photonics layer were to be processed at the top of the stack, integration with electronics defined at the frontend would require the photonics layer to adopt only low temperature processes which would not adversely affect prior processes and cause for example, dopant migration and melting of copper. High temperature grown silicon nitride therefore possesses some limitations in the range of electronic circuits which they may be integrated with. With the advancements made in ultra-low loss deuterated silicon nitride reported by Bose et al., this limitation is lifted, thus availing a wider range of implementations of process flows and realizable optoelectronic circuits. In addition to its potential impact on manufacturing, it will be interesting to see the pace at which ultra-low loss SiN grown with deuterated precursors will advance applications enabled by ultra-high-Q resonators or ultra-low loss waveguides.
